# Fabrication of High-Quality Straight-Line Polymer Composite Frame with Different Radius Parts Using Fiber Winding Process

**DOI:** 10.3390/polym13040497

**Published:** 2021-02-05

**Authors:** Jaroslav Mlýnek, Seyed Saeid Rahimian Koloor, Tomáš Martinec, Michal Petrů

**Affiliations:** 1 Department of Mathematics, Faculty of Science, Humanities and Education, Technical University of Liberec, Studentská 2, 461 17 Liberec, Czech Republic; jaroslav.mlynek@tul.cz; 2 Institute for Nanomaterials, Advanced Technologies and Innovation, Technical University of Liberec, Studentská 2, 461 17 Liberec, Czech Republic; tomas.martinec@tul.cz (T.M.); michal.petru@tul.cz (M.P.)

**Keywords:** polymer composite frame, winding of fibers, winding angle, mathematical model, straight-line composite frame

## Abstract

The extraordinary features of fibrous composites enable advanced industries to design composite structures with superior performance compared to traditional structures. Composite frame structures have been designed frequently as components of mechanical systems to resist lateral and gravity loads. The manufacturing of high-quality composite frames depends primarily on the accurate fiber winding on frames with different pro-files and curved shapes. The optimal fiber winding process on a nonbearing composite frame with a circular cross-section is described in previous works by the same authors. As an extension to that, this study focuses on the manufacturing of straight-line composite frames with different profile radii at multiple locations. Such production procedure allows continuous winding of fibers gradually on individual parts of the frame and generally with different angles of fiber winding. The winding procedure is performed using fiber-processing head and industrial robot. The procedure for calculating the distance of the winding plane of fibers on the frame from the guide-line of the fiber-processing head is targeted. This distance depends on the required angle of fiber winding, the radius of the frame, and the geometric parameters of the fiber-processing head. The coordination of the speed of winding the fibers on the frame and the speed of the passage of the frame through the winding head is also considered. Determining the correct distance of winding the fibers from the corresponding guide-line of fiber-processing head and right coordination of the winding speed and the speed of passage of the frame through the fiber-processing head ensure compliance of the required angles of fiber windings on the frame and homogeneity of winding fibers, which are the two of the most important prerequisites for producing a quality composite frame. The derived theory is well verified on a practical experimental example.

## 1. Introduction

In the past several decades, the revolution in the design of new advanced structures has forced many industries to produce structures of mechanical systems using composite materials with superior properties to replace the classic material like metal, wood, etc., [1,2]. The main advantage of using composites lies in their low weight, flexibility, and strength, weather resistance, long life, maintenance-free, etc., [1,3,4,5]. The polymer composite frames occupy an important place in the use of composites in production technologies. In particular, they are used in the aerospace industry (for example, to reinforce the airplane fuselage, or to attach the window frame to the fuselage) [6,7], in the automotive industry (for example, to reinforce the car chassis, or to consolidate the body panels, doors, and trunk) [8], in oil and gas application for the design of pressure vessels, pipelines, etc., [9,10,11], in production of agricultural machinery [12], in the production of sports equipment [13,14], and in healthcare [15,16], etc. A very important example of composite frames could be the new generation of the frame, in the form of thermoplastic composite pipe for gas transport in the offshore industry, to counter the inherent drawback of steel such as corrosion, fatigue, and weight [17,18,19].

**Figure 1:**
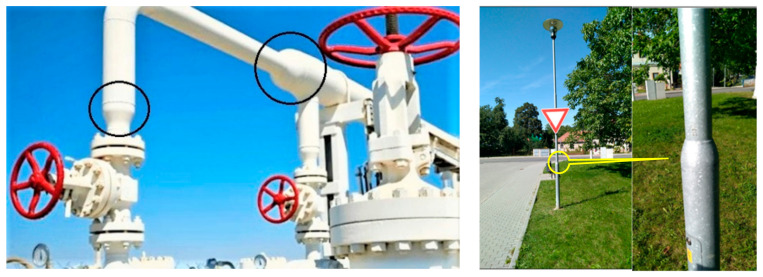
Example of frame structures in the form of (**left**) pipe structure for oil and gas application, as well as (**right**) road and street light poles.

Much research has been conducted on the manufacturing processes of composite structures to increase the structural stability and integrity [1,20]. In this regard, production of a higher quality composite frame with exceptional properties was found to be directly dependent on the quality of a homogenized and continuous fiber winding pattern [1,21,22,23]. Previous researches have shown that any inconsistency in the fiber winding pattern would result in the fabrication of large defects and voids that causes the frame structure to experience stress concentration phenomena and early damage at the defect region [14,15,16,17]. The composite frames are designed in various forms with complex hollow configurations (depending on the industrial application). Examples of the composite frame (following the objective of this research) in the oil and gas industry in the form of composite pipelines, as well as a simple street light pole, are shown in Figure 1. Many methods could be used for the manufacturing of composite frames [24], including robotic filament winding technique [1,25], pultrusion molding [26], resin transfer molding [27], braiding technique [28], etc. Nowadays, the manufacturing of polymer composite frames is promoted using industrial robots that enables fast and high-quality production of the frames [1,29,30]. Robot capability in accurate and fast fiber winding process is seen as the advantage of this technique. In robot winding, the fiber is wounded onto a nonbearing core frame with any type of complex geometries and profiles, which later is used to fabricate the composite frame through an injection molding process [1,25,29].

**Figure 2:**
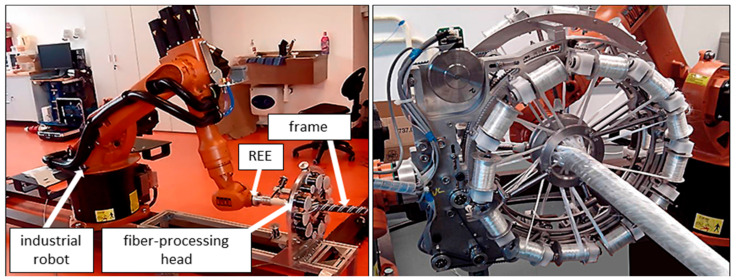
The process of winding of fibers on a frame; fiber-processing head only with one guide-line creates one layer of fibers on a frame (on the (**left**)), detail of fiber-processing head with three guide-lines that creates gradually three layers of glass fibers on the frame (on the (**right**)).

Among many types of composite frame profiles, the cylindrical frame is frequently used in many applications due to its higher mechanical toughness, and also easier manufacturing process [1,9,10,24,31]. In this regard, new investigations to advance the production technology of frame with complex profile geometries led to the design of the high-quality frame with 3D cylindrical straight and curved shapes [1,32,33,34]. In the robot winding process of the cylindrical frame, a nonbearing core frame with a circular cross-section is fastened to the working arm of the industrial robot, in which the core passes through the fiber-processing head. The movement of the frame through the head is based on the movement of the robot’s working arm. Three layers of fibers (i.e., carbon, aramid, or glass fibers) are usually wound homogeneously by this way on the core frame to ensure the consistency of the fiber arrangement. Figure 2 shows the process of fibers winding on a straight-line frame using an industrial robot and fiber-processing head.

There are many concerns in the manufacturing process of the high-quality composite frames that have been investigated in the past years [35,36,37,38,39,40]. The effect of winding patterns in filament winding of composite cylinder was studied by Azevedo et al. [37]. The number/sequence of fiber layers wound at specific angles on the composite frame are intended in the design step, in which the robot winding angle for each fiber layer is specified to ensure such arrangement in uniform, consistent, and homogeneous form (due to the anticipated stress of the composite) throughout the frame [1,21,41]. In another work, Polini et al. studied the speed and tension of winding during robotic filament winding as an important parameter that affects the mechanical properties of the composite [36]. In another work, the high-speed fiber placement technology was proposed as a new methodology of motion planning for redundant robotic system [39], to solve the time optimization problem of robot motion [39,40,42]. Mlýnek et al. described an optimized winding process that was considered for the case of a generally 3D geometrically shaped frame having a constant circular cross-section of the frame [1,23]. The optimization, and also the control of robot trajectory in the fiber winding process, has been the topic of many studies for the accurate fabrication of composite frames [1,35,40,42,43,44,45,46,47].

Although several studies have been performed on the optimization of the fiber winding process in the high-quality production of the composite frames, the choice of the frame in most cases was a fixed cross-sectional profile. While composite frames are often produced in several parts with different radii of circular cross-section (for example circled section shown in Figure 1), in this case, it is supposed, each part of the frame is wound with different winding angles. Owing to the complexity of the profile shape, the variation of the cross-sectional radius causes a concentration zone that intensifies the stress quantity and the possibility of failure; therefore, accurate fiber winding of that region will be a great concern. So far, the production of frame composites with different cross-sections and different winding angles has not been carried out to a greater extent, the main reason being difficult technical feasibility. In this regard, this study presents a procedure enabling the optimization of the winding process of fibers of the nonbearing straight-line frame with a few parts with different radii of circular cross-section and, thus, ensuring the production of a high-quality composite frame for such types of frames. The process of winding fibers on a straight-line frame uses an industrial robot and a fiber-processing head, which are shown in Figure 2. During the production of the composite frame, after winding the prescribed layers of fibers on a nonbearing core frame, the core is inserted into a preheated mold and then the matrix is injected into the mold under controlled pressure and temperature. A detailed description of the frame curing process is described in [25,29].

## 2. Manufacturing of Polymer Composite Frame

Detailed information about the manufacturing of the polymer composite frame is provided in previous work by the same authors [1], which include the optimization of the fiber winding process on a frame based on finding the optimized off-line trajectory of the industrial robot. This procedure ensures the correct fiber winding angles of the individual layers and the homogeneity of the fiber windings. In this chapter, we will focus on extending the optimized fiber winding process to the case of a straight-line frame containing several parts with different radii of circular cross-sections. At the same time, it will be possible to wind the fibers on individual parts of the frame at different winding angles.

**Figure 3:**
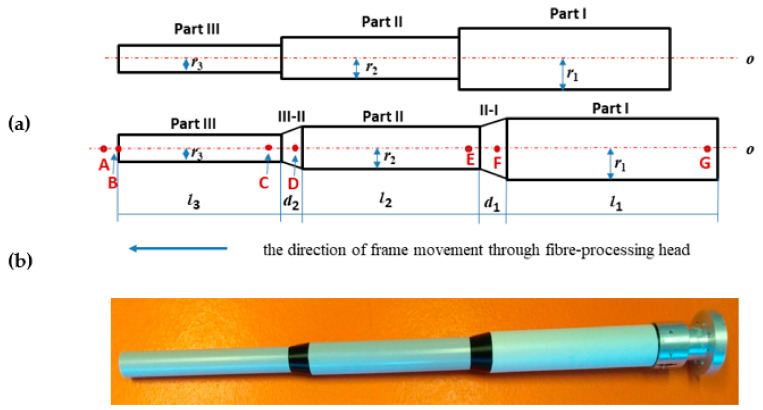
(**a**) Example of a longitudinal cross-section of a straight frame with three parts with different radii, jump transition between individual parts of the frame ((**a**) upper), continuous transition between the individual parts of the frame ((**a**) lower), and (**b**) test non-bearing polyurethane frame, continuous transition between the individual parts of the frame were created on a 3D printer. The metal part adjacent to the frame (in the right part of the picture) is used to attach the frame to the robot-end-effector (REE).

Adherence to the correct winding angles along the entire length of the frame is not feasible in the case of a step transition between parts of the frame with different cross-sectional radii (see Figure 3a (above)). It is technically impossible to ensure a smooth winding of the fibers in the transition part. A necessary prerequisite for ensuring optimized fiber winding is a continuous (not stepwise) transition between each individual parts of the frame with different radii (see Figure 3a (bellow)).

**Figure 4:**
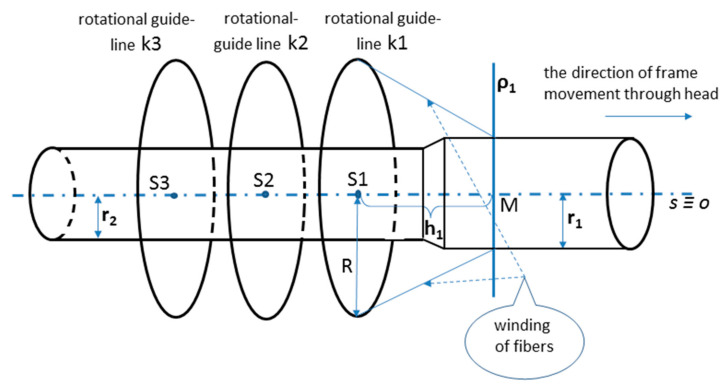
Diagram of the passage frame through the fiber-processing head with three guide-lines. For the first guide-line k1, the plane of fibers winding onto the frame is marked $\rho_{1}$, which is perpendicular to the axis *s* of the winding head (and in this case also to the axis *o* of the frame).

Therefore, considering the information provided in previous work, Mlynek et al. [1], this study deals only with the continuous transition between parts with different radii. In this regard, a fiber-processing head with three guide-lines (12 spools of fibers are attached to each ring) with the same radius (see Figure 2 on the right). The frame is attached to the end of the robot arm (robot-end-effector (REE), see Figure 2 on the left and Figure 3b) and passes through the fiber-processing head on the base of the movement of the working arm of the robot. Each of the three guide-lines provides, gradually, the formation of a layer of fibers on the frame. Each layer of fibers is generally wound under different angles. Since we solve the task of winding a straight frame, the trajectory of the robot (exactly trajectory of REE) is also straight and, therefore, the task of its optimization is eliminated. To ensure the correct winding angle of the fibers on each part of the frame with a given radius (see Figure 3a (bellow)), we need to know the distance of the plane of winding fibers on the frame from the corresponding guide-line of the head. This plane is perpendicular to the axis *s* of the head (see Figure 4, distance plane of winding $\rho_{1}$ from corresponding guide-line k1 is value *h*1= ‖S1M‖, fiber winding planes $\rho_{2}$ and $\rho_{3}$ are determined analogously for guide-lines k2 and k3).

The constant correct ratio of the rotational angular speed of guide-line of fiber-processing head and speed of passage of the frame through the fiber-processing head is the second important condition to ensuring the correct winding angle.

The procedure for calculation of the distance of the winding plane from the corresponding guide-line and a constant ratio assurance procedure of rotational angular speed of guide-line and speed of the frame during the passage through the head are described in the following sections.

### 2.1. Determination of the Winding Plane

**Figure 5:**
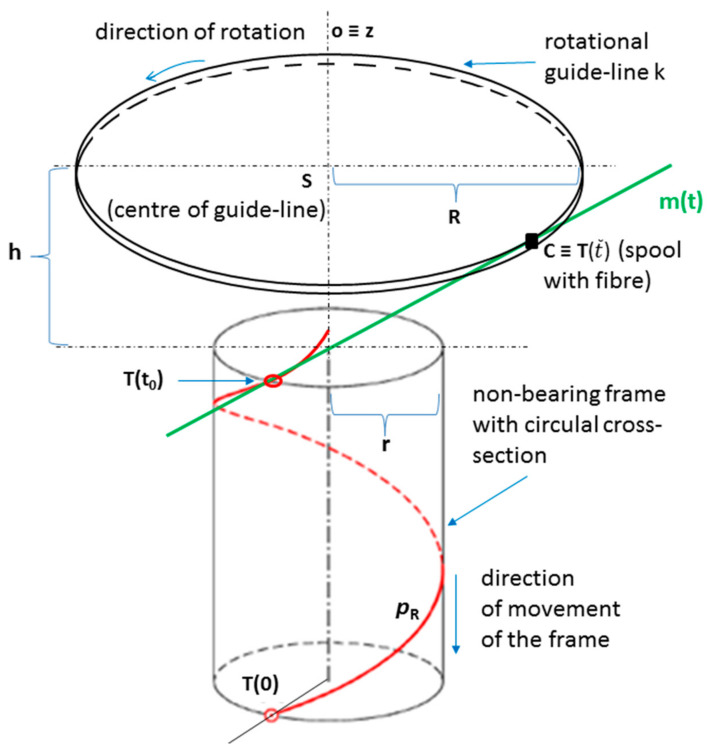
Schematic representation of the winding of a fiber by means of a rotation guide-line *k* of the fiber-processing head on a frame with a circular cross-section.

**Figure 6:**
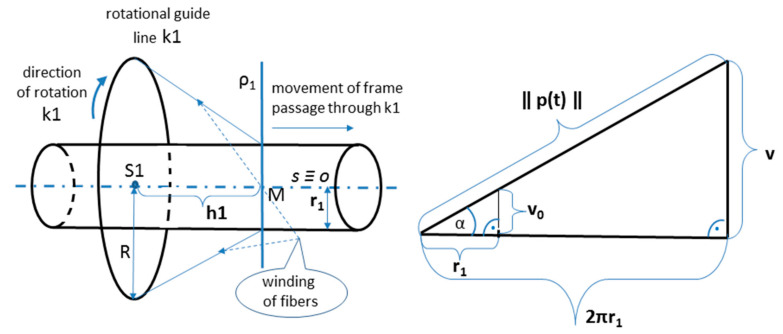
Schematic representation of the passage of the frame through the rotating guide-line k1, the plane of the winding of the fibers ρ_1_ and the distance h1 of the plane from the guide-line (on the left), h1 = ‖S1M‖. Characteristic triangle of the helix on the right (r_1_—radius of the helix, v_0_—reduced pitch, v—the pitch of the helix, α – angle slope that is referred to as winding angle, ǁǁp(t)ǁǁ—length of helix *p*(t) for t∈〈0, 2π〉) (on the right).

We assume a common central axis *o* of the fiber-processing head and a straight-line frame passing through the fiber-processing head during the winding process (see Figure 4). The winding process from the geometric point of view is shown schematically in Figure 5. Each wound fiber creates helix on the frame surface. The common central axis *o* of the frame (and, thus, also the axis of the helix) on schematic Figure 5 is identical to axis *z* of 3D right-handed Euclidean space E_3_. The distance of the rotational guide-line *k* of the fiber-processing head from the plane of fiber winding (element of this plane on Figure 5 is the cross-section of the frame containing point *T*(*t*_0_)) depends on the radius *R* of the rotational guide-line *k*, the radius *r* of wound part of the frame (each part of the frame has circular cross-section) and required angle α of winding of the fiber on the frame. We suppose the fiber is wound on the frame as a right-handed helix *p_R_* (the second option would be left-handed helix) with axis *o* ≡ *z*, helix radius *r* (radius of the considered part of the frame). We consider the angle of winding of the fiber onto the surface of the frame to be the angle slope α of the corresponding helix (see Figure 6 on the right and in detail [48], Chapter 2). However, technicians in the manufacture of composite frames often call δ the angle of winding of the fibers onto the frame (see Figure 6 on the right), where

(1) $$\delta \;=\;\frac{\pi }{2}-\alpha .$$

We assume $\alpha \in \left(0,\;\frac{\pi }{2}\right)$. The case α=0 would mean a constant winding of the fiber on the frame in the circle in the plane in which the guide-line *k* lies from which the fiber is unwound. The case $\alpha =\frac{\pi }{2}$ would mean laying the fiber on the surface of the frame parallelly to the axis *o* of the frame.

We use the homogeneous form of expression of vectors and points in E_3_ (the fourth coordinate of the point is 1 and of the vector is 0), in which the detail is presented elsewhere [48]. Then the parametric equation of helix *p_R_* can be expressed in the form of $p_{R}\left(t\right)=\left(r\cos t,\;r\sin t,\;v_{0}t,\;\;1\right),t\in <0,\;\infty )$ (see Chapter 2 in [48]). Parameter $v_{0}$ determines reduced pitch of helix (length of translation during rotation of fiber by one radian, $v_{0}=r.\mathrm{tg}\alpha$), see also Figure 6 on the right. Then derivative $p_{R}^{\prime }\left(t\right)=\left(-r\;\sin t,\;r\cos t,\;v_{0},\;0\right)$ (see Chapter 2 in [48]). Equation of tangent $m\left(t\right)$ of helix *p_R_*(*t*) at point $T\left(t_{0}\right)=\left(r\cos t_{0},\;r\sin t,\;v_{0}t_{0},\;\;1\right)$ for given *t*_0_ is defined in form

(2) $$m\left(t\right):\left(x,\;y,\;z,\;1\right)=\left(r\cos t_{0},\;r\sin t_{0},\;v_{0}t_{0},\;1\right)+\left(-r\sin t_{0},\;r\cos t_{0},\;v_{0},\;0\right)t.$$

We determine the intersection *C* of tangent *m*(*t*) that is defined by relation (2), and the shell of the cylindrical surface with axis *o* and radius *R* (see Figure 5). The spool with fiber that creates helix *p_R_* during the winding process is represented by point C. This point C is the element of the cylindrical surface with central axis *z* and radius *R*. For each point A = [x_A_, y_A_, z_A,_ 1] of considered cylindrical surface, it is true $x_{A}^{2}+y_{A}^{2}=R^{2}$. At the same time, point C lies on tangent *m*(*t*). Therefore, exists the real value $\tilde{t}$ the equation holds

(3) $${\left(r\cos t_{0}-\tilde{t}r\sin t_{0}\right)}^{2}+{\left(r\sin t_{0}+\tilde{t}r\cos t_{0}\right)}^{2}=R^{2}$$

From Relation relation (3), we calculate the value of $\tilde{t}$ after making simple algebraic adjustments, $\tilde{t}=\sqrt{\frac{R^{2}-r^{2}}{r^{2}}}$.

Distance *h* of the rotational guide-line of the fiber-processing head from the plane of fiber winding on the frame is then given by the difference of the *z*-th coordinates of point $C\equiv T\left(\tilde{t}\right)=\left[rcost_{0}-\tilde{t}rsint_{0},\;rsint_{0\;}+\tilde{t}rcost_{0\;},\;v_{0}t_{0}+v_{0}\tilde{t},\;1\right]$ and point $T\left(t_{0}\right)$. We use relation $v_{0}=r\cdot tg\alpha$ (also see Figure 6 on the right) to derive distance *h*, and we gradually receive

$$h=z_{T\left(\tilde{t}\right)}-z_{T\left(t_{0}\right)}=v_{0}t_{0}+v_{0}\tilde{t}-v_{0}t_{0}=v_{0}\tilde{t}=v_{0}\sqrt{\frac{R^{2}-r^{2}}{r^{2}}}=r\cdot tg\alpha \cdot \sqrt{\frac{R^{2}-r^{2}}{r^{2}}}=tg\alpha \cdot \sqrt{R^{2}-r^{2}}.$$

Thus, distance *h* is given by the relation (4)

(4) $$h=tg\alpha \cdot \sqrt{R^{2}-r^{2}}.$$

We, therefore, determined the distance of the rotational guide-line *k* with radius *R* from the corresponding winding plane for radius *r* of the cross-section of the winding part of the frame and for the specified winding angle α. We would similarly determine distance *h* for a negative value of angle α. It would be a left-handed helix in this case. However, the distance will be the same for both cases.

The distance of the winding plane of the fibers from the corresponding guide-line of the fiber processing head depends on the required winding angle α, the radius *R* of the head, and the radius *r* of the straight frame. Calculation of the distance is given by relation (4). Table 1 shows the distances of the guide-line from the winding plane depending on the different input parameters in relation (4). The table shows, in accordance with equation (4), that distance *h* increases with increasing values of winding angle α, radius *R* of guide-line, and distance *h* decreases with increasing value of radius *r* of the frame.

Distance *h* of the fiber winding on the frame from the corresponding guide-line can be calculated using relation (4). However, to maintain the correct angle of winding of the fiber on the frame, a mutual correction of the speed of frame passage through the guide-line and the guide-line angular rotational speed have to be ensured. The fulfillment of this condition is solved in the following section.

### 2.2. Controlling of the Speed of Fibers Winding

In this part of the article, we derive the interrelation between the speed of passage of the frame through the guide-line of the fiber-processing head and the angular rotation speed of the guide-line during the winding process. As we stated in Section 2.1, each wound fiber creates helix on the frame surface. The pitch of helix *v* is defined by relation (see Figure 6 on the right, in detail see [48], Chapter 2)

(5) v=2πr·tgα,

where *r* denotes radius of the helix (in our case radius of the frame) and α denotes the angle slope of the helix (in our case winding angle of fiber onto the frame). Size *v* grows with increasing values *r* and α (we recall $\alpha \in \left(0,\;\frac{\pi }{2}\right)$). The pitch of helix v defined by relation (5) indicates, that frame moves in the direction of the frame axis *o* (this axis is identical to axis *s* of the fiber-processing head, see Figure 4 and Figure 6 on the left) by a distance *v* after one turn of the guide-line (i.e., during the formation of one thread of helix).

Now, we also focus on the rotational motion of the guide-line. Peripheral speed *u* of guide-line tells us how large an arc creates point lying on the guide-line per one second. Angular speed ω of guide-line expresses the traversed angular path in an arc measure (in radians) per one second. In general, the relationship applies [49]:

(6) u=ω·R,

where *R* denotes the radius of the guide-line. Let us denote the speed of uniform straight movement of the frame as *w*. We require the frame to make path *v* for the same amount of time as the point on the circumference of a guide-line circle makes path 2πR. We consider the even movement of the frame and the guide-line. Then we can use relation (5) and write $\frac{u}{w}=\frac{2\pi R}{v}=\;\frac{2\pi R}{2\pi r.tg\alpha }=\;\frac{R}{r.tg\alpha }.$ We obtain by using relation Equation (6) $\frac{\omega R}{w}=\;\frac{R}{r\cdot g\alpha }$.

We express angular speed ω from this relation and obtain

(7) $$\omega =\;\frac{1}{r\cdot tg\propto }\cdot w.$$

In Relation (7), the value of angular speed ω [rad/s] of guide-line depends on values of variables speed of movement of frame *w* [m/s], radius *r* [m], and winding angle α [rad]. The parameter *w* is known and is usually a constant value when the fibers are wound on the frame. Values of angular speed ω depending on the parameters in Relation (7) are listed in Table 2.

It follows from Relation (7) and from Table 2 that the value of angular speed ω is growing with increasing speed movement *w* and is slower with increasing frame radius *r* and fiber winding angle α.

#### Note

During the winding process, a nonbearing frame (usually from polyurethane) is connected to the end of the robot working arm, specifically to REE. Movement frame speed *w* in each partial passage is known (a constant speed is assumed). We can specify rotational angular speeds of three rotational guide-lines by three external axes e1, e2, and e3 within Tool Centre Point (TCP) of the industrial robot (TCP defines primarily actual position of REE). Three external axes of industrial robot specify rotational angle speeds ω of each rotational guide-line (k1, k2, and k3; see Figure 2 on the right and Figure 4) given by Relation (7). This way, the correct winding angle α of fibers on the frame is ensured for each of guide-line k1, k2, and k3 (see Figure 4).

#### General Procedure

At the end of this section, we present a procedure for winding of a layer of fibers on the frame using one guide-line of the fiber-processing head. We determine the required values for the transition of winding from Part I of the frame with a given radius of the cross-section to another subsequent Part II of the frame with a different radius of the cross-section. We give a general example of the procedure for calculating of required values of fiber winding distances from the guide-line and guide-line angular velocities when winding at specified angles needed to make the correct winding. We assume a constant speed *w* of the passage of the frame through the fiber-processing head.

1. Determination of the distance h1 of the fiber winding on Part I of the frame from guide-line (use Relation (4)).
2. Calculation of angular speed ω1 needed to ensure the required angle of the fibers winding on Part I (use Relation (7)).
3. Determination of distance h2 of the fiber winding on Part II of the frame from guide-line (use Relation (4)).
4. Calculation of angular speed ω2 needed to ensure the required angle of winding of the fibers on Part II (use Relation (7)).

Then, we assume that the winding process is in progress. The transition of the winding process from Part I to Part II is described in the following steps.

5. Termination of angular speed ω1 of guide-line in distance h1 before the end of Part I when the frame goes through the guide-line.
6. Smooth transition from angular velocities ω1 to ω2 of guide-line.
7. Starting angular speed ω2 of guide-line, when the beginning of Part II is in distance before guide-line h2.

If three layers of fibers are wound at the same time, we apply the above procedure for each of the three guide-lines of the fiber-processing head. The angular speed ω of the individual guide-lines are independent of each other.

The use of the described procedure as “winding a frame with several parts with different cross-sectional radii” is described in a practical example in the following chapter.

## 3. Results and Discussions

In this section, the focus is on the verification and use of theoretical knowledge derived in the previous section.

### 3.1. Verification Example

We consider the straight-line nonbearing frame that consists of three parts with a circular cross-section, each part having a different radius of cross-section, see Figure 3a (below) and Figure 3b. The smooth transition of the frame surface is made between the individual parts of the frame. In the case of a “jump transition” between two frame parts with different radii, it would not be possible to make a correct winding of fibers on the frame, see Figure 3a (above). Radii of Parts I, II, and III of the frame are gradually *r*_1_= 40 [mm], *r*_2_ = 30 [mm], *r*_3_ = 20 [mm]. The lengths of the individual parts are *l*_1_ = *l*_2_ = *l*_3_ = 500 [mm]. Radius R of the guide-line of the fiber-processing head is R = 50 [mm] (see Figure 6 on the left). In this article, we consider, generally, a fiber-processing head with three guide-lines k1, k2, and k3 (see Figure 2 on the right, Figure 4). Three layers of fibers are gradually formed during the passing of the frame through the fiber-processing head. However, for clarity, we consider only winding of one layer using guide-lines k1 (see Figure 6 on the left) in this, our verification example. Part I will be wound under angle $\frac{\pi }{4}$, Part II under angle $\frac{\pi }{6}$ and Part III under angle $\frac{\pi }{3}$. The winding angles on the transition Part II-I and Part III-II, respectively, will be smoothly changed from angle $\frac{\pi }{6}$ to angle $\frac{\pi }{4}$ and from angle $\frac{\pi }{3}$ to angle $\frac{\pi }{6}$, respectively. Lengths *d*1 and *d*2 of both transition Parts II-I and III-II (see Figure 3a (bellow)) are the same value *d*1= *d*2= 80 [mm]. We suppose a constant speed *w* = 50 [mm/s] passage of the frame through guide-line *k*1 of the fiber-processing head.

The end of Part I of the frame is attached to the REE (see Figure 3b) and the frame passes through the guide-line k1 of the fiber-processing head firstly by Part III, then by Part II and finally by Part I. We calculate the distance *h*3 of the winding plane from the guide-line using the relation (4) and rotational angular speed ω3 ensuring the required winding angle $\alpha 3=\frac{\pi }{4}$ by applying relation (7) for Part III. We determine analogously values of *h*2 and ω2 for Part II and *h*1 and ω1 for Part I. Note that winding angle α is given in relation (4) and relation (7) in radians. The parameter values are summarized in Table 3.

Distance *h*1 of winding plane $\rho_{1}$ from the guide-line *k*1 is equal to the distance of points *S*1 and *M* in Figure 6 on the left. During the winding process, the frame gradually passes through the winding head. We remind, Part III goes first through the head, then Part II, and finally Part I. Following Table 4 shows the marked points on the axis *o* of the frame (axis *o* and axis *s* of the fiber-processing head are identical, *o* ≡ *s*). Values of points on the axis *o* of the frame (see Figure 3a (below)) increase from left to right. Marked points on the axis *o* with increasing values gradually pass through the guide-line *k*1. Table 4 describes the winding activity in the moment, when the individual points in the left column of the table pass through guide-line k1. Corresponding angular speeds ω of *k*1 to the passing points of the *o*-axis through guide-line *k*1 are also given in Table 4.

The frame passes through guide-line *k*1 of constant speed *w* = 50 [mm/s]. Therefore, we can determine the passage times of individual points listed in the left column of Table 4 through guide-line k1. We can define the rotational angular speed of k1 by external axis e1 within TCP of industrial robots. We use this procedure to ensure the required angle of winding of fibers on the parts Part III, Part II, and Part I. An even transition of the angular velocity between the individual parts of the frame with different radii is also ensured by using the external axis e1 within TCP. The layer of wound fibers by k1 is formed by the above method.

In the case of three wound layers of fibers on the frame being created, the first layer is wound using guide-line k3, then the second layer using guide-line k2, and finally the layer created by guide-line k1 (see Figure 2 and Figure 4 on the right).

**Figure 7:**
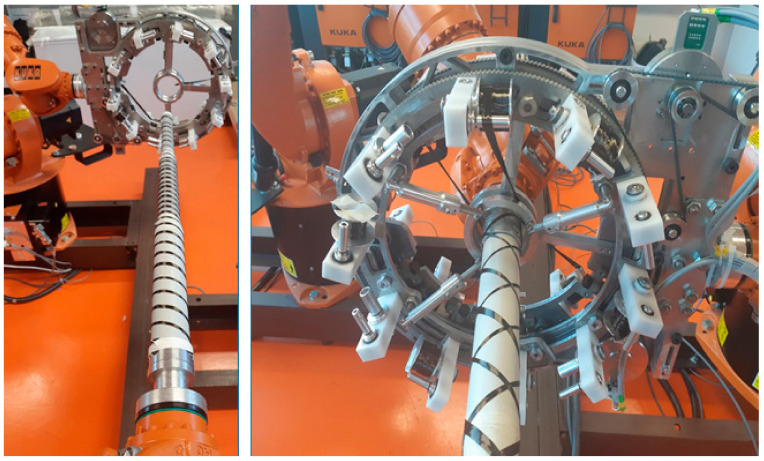
The resulting winding of one fiber on the composite frame of this section using only one guide-line, one carbon fiber (for clarity), and an industrial robot (on the (**left**)), see the frame in Figure 3b. Passage of the composite frame through the fiber-processing head using one guide-line and only two carbon fibers and the industrial robot (on the (**right**)).

The final winding of one carbon fiber on the composite frame of verification example, in accordance with Table 1 is shown in Figure 7 on the left.

**Figure 8:**
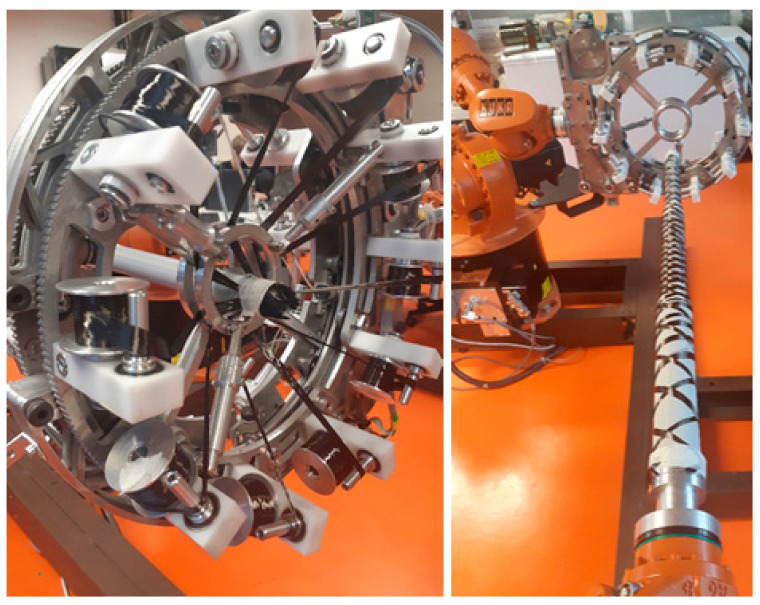
Detail of guide-line of the fiber-processing head containing spools with fibers before the start of winding (on the left). The resulting winding of two carbon fibers on the composite frame using two guide-lines (first guide-line winds one fiber under angle $\frac{\pi }{4}$, second winds one fiber under angle $-\frac{\pi }{4}$ on three parts of the frame with different radii of cross-section (on the right).

The detail of the guide-line of fiber-processing head and coils with carbon fibers is shown in Figure 8 on the left. The resulting winding of two fibers by two guide-lines is shown in Figure 8 on the right.

### 3.2. Composite Frame Production

The described fiber winding technology in the case of a straight-line polymeric composite frame with several parts and different cross-sectional radii makes it possible to ensure the correct angles of winding the fibers onto the frame and also the homogeneity of the winding of the fibers onto the frame. Ensuring the correct winding of fibers on the frame (i.e., ensuring the correct angle of the fiber winding and the homogeneity of the fiber winding) is one of the most important prerequisites for the production of high-quality composite frames. We can wind three layers of fibers on the frame using the fiber-processing head with three guide-lines in one pass of the frame by the head. Each layer of fibers is wounded by one guide-line. If it is necessary to wind more layers of fibers, it is possible to repeat the frame passage through the fiber-processing head. The procedure described in Chapters 2 and its verification in Section 3.1 makes it possible to perform a precise winding of the fibers on a composite frame with different parts of the cross-sectional radius at the specified winding angles on these parts. Making precise windings of fibers at a specified angle and ensuring the homogeneity of the windings of a given layer of fibers provides the use of Relation (4) to calculate the distance of the winding (of fibers on the frame) from the corresponding guide-line and use of Relation (7) to calculate the actual required angular speed of corresponding guide-line.

The winding process of fibers onto the composite frame using only one guide-line and two carbon fibers (only two spools located on the guide-line are used) is shown in Figure 7 on the right. The use of the two fibers is implemented to make a clearer winding of the fiber.

**Figure 9:**
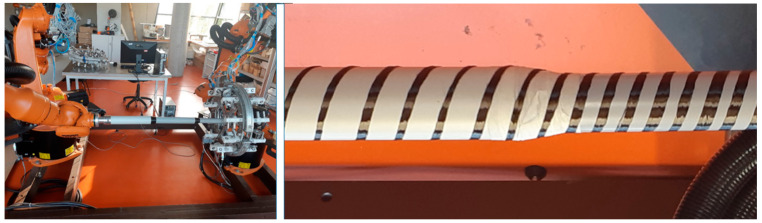
The attachment of the frame to REE and the fiber-processing head before starting the winding process (on the left). Fiber winding density for constant winding angle α = $\frac{\pi }{6}$ and frame parts with different cross-sectional radii r_1_ = 30 [mm], r_2_ = 20 [mm] (on the right).

The attachment of the verification frame from Section 3.1 and the fiber-processing head before starting the winding process itself is shown in Figure 9 on the left.

#### Note

The density of winding the fiber onto the frame at constant guide-line radius R = 50 [mm] and the radii of the circular cross-sections of the parts of the frame are r_1_= 30 [mm], r_2_ = 20 [mm] and prescribed winding angle α= $\frac{\pi }{6}\;$(as mentioned above, technologists of composite production usually call the winding angle δ = $\frac{\pi }{2}-\;\alpha$) is higher in the case of winding a frame part with a smaller cross-sectional radius. The ratio of the winding density on both parts of the frame is given by the ratio of pitch of helix v_2_ for part of the frame with radius r_2_ and pitch of helix v_1_ for part of the frame with radius r_1_ (see the relation (5) and Figure 6 on the right), $\frac{v_{2}}{v_{1}}=\frac{tg\alpha \;2\pi r_{2}}{tg\alpha \;2\pi r_{1}}=\frac{r_{2}}{r_{1}}=\frac{2}{3}$. It means that on the part of the frame with radius r_2_, the winding of the fiber is denser in proportion 3:2 (see Figure 9 on the right).

We suppose adjacent parts of the frame with different radii have a continuous transition. Using the methods of winding the fibers on the frame mentioned in this article, it allows the following windings of fibers on the composite frame to be performed:

1. realization of simultaneous winding of three layers of fibers on the frame at different angles,
2. ensure the winding of individual parts (including parts with different radii of their cross-sections) of the frame at specified different angles for given winding layer,
3. possibility of ensuring the same winding angle for each winding layer for all parts of the frame with different radii of their cross-sections,
4. adjacent parts of the frame with different radii have a continuous transition of wound fibers for a given layer.

Relations (4) and (7) have not been published yet and are part of the original development of frame composite winding technology by the authors of this article. A new procedure for winding fibers on a frame composed of several parts with different cross-sectional radii is given in the article for a straight frame. Mlynek et al. [1] solved the optimization problem of fiber winding in the case of a frame composite, generally 3D geometrically shaped. A differential evolution algorithm is used during the optimization process. The result of the research provided in Mlynek et al. [1] in combination with the content of this study, which focuses on the case of general 3D geometrically shaped frames with different cross-sectional radii at multiple locations, could be a suitable topic for further development of frame composite winding technology.

## 4. Conclusions

Based on the procedure in this article, the correct angle of the fiber windings and the homogeneity of the fiber windings on the frame are ensured. This article solves the problem of right winding of fibers on the straight-line frame with a few different radii of their cross-sections. It is, therefore, an extension of the problem solved by Mlynek et al. [1]. The three layers of fibers are parallel wound as the frame passes through the fiber-processing head. The winding of each layer of fibers is ensured by one guide-line of the fiber-processing head. The procedures described in the article allow the following steps to be performed before starting and subsequent realization of the fiber winding process onto the straight-line frame composed of several parts with different radii of their cross-sections. Therefore, it is possible to:

- Calculate the distance of winding the fibers (on the frame) from the corresponding guide-line. This distance depends on the size of the required winding angle, the radius of the guide-line, and the radius of the relevant wound part of the frame.
- Determine the angular speed of the guide-line so that the fibers are wound at the desired angle. Angular speed depends on the defined winding angle, the radius of the relevant part of the frame, and the constant size of passage speed of the frame through the head.
- Based on the previous two points and assuming a constant speed of passage of the frame through the head, we can determine:
- when and with what angular speed is started and ended winding by guide-line so that the fibers are wound at a desired angle on a specified part of the frame;
- the continuous transition between two individual parts of the frame with different radii is wound at a gradually varying angle from the finished winding angle to the next desired winding angle on the following part of the frame, while the gradual change of winding angle is performed based on the continuous change of angular speed of guide-line;
- the continuous change of winding angle on a straight-line frame with a constant cross-sectional radius;
- the same winding angle on the parts of the frame with different radii of the cross-section.

The control procedure of rotational angular speed of guide-line of the fiber-processing head in conditions of the constant passage of the frame through the fiber-processing head is realized using external axes of the industrial robot.

The procedures described in this article enable optimal (accurate) winding of several layers of fibers at specified winding angles on individual parts of the frame with different radii of their cross-sections. The outputs of this article enable the expansion of the production of some special frame composites, which can replace the production of the necessary components from classic materials. The development of these composite frames is increasingly required due to the frequent different stresses of the individual parts of the composite in their practical use.

## Figures and Tables

**Table 1:** Calculation of distance winding plane from guide-line depending on radius R of guide-line, frame radius r, and the required size of winding angle α.

R [mm]	r [mm]	α [º]	δ [º]	h [mm]
50	20	30	60	26.4575
		45	45	45.8258
		60	30	79.3735
	30	30	60	23.0940
		45	45	40.0000
		60	30	69.2820
	40	30	60	17.3205
		45	45	30.0000
		60	30	51.9615
80	20	30	60	44.7213
		45	45	77.4597
		60	30	134.1641
	30	30	60	42.8174
		45	45	74.1620
		60	30	128.4524
	40	30	60	39.1000
		45	45	69.2820
		60	30	119.1000

**Table 2:** Calculation of guide-line angular speed ω of depending on parameters in relation (7).

w [mm/s]	r [mm]	α [º]	tgα	ω [rad/s]
25	20	30	0.5774	2.1649
		45	1	1.2500
		60	1.7321	0.7217
	30	30	0.5774	1.4433
		45	1	0.8333
		60	1.7321	0.4811
	40	30	0.5774	1.0824
		45	1	0.6250
		60	1.7321	0.3608
100	20	30	0.5774	8.6600
		45	1	5.0000
		60	1.7321	2.8867
	30	30	0.5774	5.7730
		45	1	3.3333
		60	1.7321	1.9244
	40	30	0.5774	4.3467
		45	1	2.5000
		60	1.7321	1.4433

**Table 3:** An overview of the calculated values of the distance *h* of the winding plane from the guide-line k1 and the rotational angular speed ω of the guide-line for individual parts of the frame. Constant speed of passage of the frame through guide-line k1 is equal of value *w* = 50 [mm/s].

R [mm]	w [mm/s]	Part I, α1 = $\frac{\pi }{4}$		
**50**	**50**	r_1_ [mm]	*h*1 [mm]	ω1 [rad/s]
		40	30,000	1250
		**Part II, α2 =** $\frac{\pi }{6}$		
		r_2_ [mm]	*h*2 [mm]	ω2 [rad/s]
		30	23,094	2887
		**Part III, α3 =** $\frac{\pi }{3}$		
		r_3_ [mm]	*h*3 [mm]	ω3 [rad/s]
		20	79,371	1443

**Table 4:** An overview of the locations of the starts and ends of fiber winding on the frame by guide-line *k*1 at the required angles.

Value of the Point Lying on Axis *o* of the Frame [mm]	Description Position of the Point	Angular Speed ω [rad/s]	Action When Point Goes Through Guide-Line k1
A = 0	corresponding point of begin of Part III is B = A+*h*3	ω3 = 1.443	start of fiber winding on Part III at angle of α3 = $\frac{\pi }{3}$
C= 500	C = A + *l*3		end of fiber winding on Part III at angle of α3 = $\frac{\pi }{3}$
D = 636,287	D = *h*3 + *l*3+ *d*2 − *h*2	ω2=2.887	start of fiber winding on Part II at angle of α2 = $\frac{\pi }{6}$
E = 1136,277	E = *h*3 + *l*3 + *d*2 + *l2* − *h2*		end of fiber winding on Part II at angle of α2 = $\frac{\pi }{6}$
F = 1209,371	F = *h*3 + *l*3 + *d*2 + *l*2 + *d*1 − *h*1	ω1 = 1.250	start of fiber winding on Part I at angle of α1 = $\frac{\pi }{4}$
G= 1709,371	G = *h*3 + *l*3 + *d*2 + *l*2 +*d*1 + *l*1 − *h*1		end of fiber winding on Part I at angle of α1 = $\frac{\pi }{4}$

## Data Availability

Not Applicable.
